# Evidence for the embodiment of the automatic approach bias

**DOI:** 10.3389/fpsyg.2022.797122

**Published:** 2022-09-09

**Authors:** Johannes Solzbacher, Artur Czeszumski, Sven Walter, Peter König

**Affiliations:** ^1^Institute of Cognitive Science, Osnabrück University, Osnabrück, Germany; ^2^Department of Clinical Psychology, Vrije Universiteit Amsterdam, Amsterdam, Netherlands; ^3^Department of Neurophysiology and Pathophysiology, University Medical Center Hamburg-Eppendorf, Hamburg, Germany

**Keywords:** approach-avoidance task, cognition, automatic approach-avoidance tendencies, automatic approach bias, embodiment, action

## Abstract

Tendencies of approach and avoidance seem to be a universal characteristic of humans. Specifically, individuals are faster in avoiding than in approaching negative stimuli and they are faster in approaching than in avoiding positive stimuli. The existence of this automatic approach-avoidance bias has been demonstrated in many studies. Furthermore, this bias is thought to play a key role in psychiatric disorders like drug addiction and phobias. However, its mechanisms are far from clear. Theories of embodied cognition postulate that the nature of gestures plays a key role in this process. To shed light on the role of the involved gesture we employed a 2 × 2 factorial design with two types of stimuli. Participants had either to approach positive and avoid negative stimuli (congruent conditions) or to avoid positive stimuli and approach negative stimuli (incongruent conditions). Further, they responded either with a joystick or a button press on a response pad. Participants reacted faster in congruent conditions, i.e., avoiding negative stimuli and approaching positive stimuli, than in incongruent conditions. This replicates the known approach and avoidance bias. However, direct analysis of the button press condition revealed no reaction time advantage for congruent trials compared to incongruent trials. In contrast, in the joystick condition participants were significantly faster performing congruent reactions than incongruent reactions. This interaction, a significant reaction time advantage, when the response is enacted by moving a joystick towards or away from the body provides evidence that approach-avoidance tendencies have a crucial bodily component.

## Introduction

Behaviours of approach and avoidance are two of the most primal and natural ways to generally act and react to encounters of any kind in our world. We naturally approach known friends when we meet them on the street and avoid obscure alleys in the night. In our everyday life we generally tend to approach certain things rather than avoiding them, mostly if we associate the encounter with something positive to happen. Likewise, we avoid certain things rather than approaching them, mostly if we expect something negative from the encounter. Those general behavioural tendencies can in certain contexts be observed in connection with psychiatric disorders like drug addiction (Ernst et al., 2014) or phobia (Rinck and Becker, 2007) and their modification is used in therapy and treatment (Hertel and Mathews, 2011; Wiers et al., 2011). Thus, understanding approach and avoidance behaviour might not only be indispensable for a better understanding of cognition in general but particularly helpful for psychological and therapy-related reasons.

One way to study and measure an individual’s tendency to approach or avoid certain types of stimuli rather than others is using what is called an approach-avoidance task. In this reaction-time-based setup individuals are instructed to either push away or pull towards them specific cues like words (Chen and Bargh, 1999) or pictures (Bradley et al., 2008) for example according to their valence (Phaf et al., 2014) as fast as they can. Here, an individual’s general tendency to approach positive stimuli rather than avoid them and to avoid negative stimuli rather than approach them is reflected in their reaction times. Similar effects have been shown for individuals suffering from substance use disorder like heavy drinkers (Wiers et al., 2009), alcohol use disorder (Wiers et al., 2011; Wiers et al., 2014), heroin use disorder (Zhou et al., 2012a), smokers (Bradley et al., 2008; Wiers et al., 2013; Mühlig et al., 2016) and cannabis users (Cousijn et al., 2011). All these groups approach drug-related cues faster than neutral cues or faster than healthy controls. But the effect extends even further: Individuals suffering from spider phobia are faster in avoiding spider-related stimuli than in avoiding neutral cues (Rinck and Becker, 2007) and similar effects hold for socially anxious people (Heuer et al., 2007) and excessive online gamers, who approach gaming-related stimuli faster than neutral cues (Zhou et al., 2012b; Jeromin et al., 2016). Healthy individuals seem to exhibit an approach bias for chocolate (Dickson et al., 2016) and are faster in approaching appetizing food compared to neutral, non-food items (Schroeder et al., 2016; Booth et al., 2018). Generally, approach-avoidance tasks show that we are faster in avoiding what we fear or dislike and in approaching what we want, like or need. This reaction-time-based effect is called an automatic approach-avoidance bias. Given that approach and avoidance tendencies seem to be a rather universal characteristic, the question is: Why do we exhibit this bias?

One way to get more insight into the mechanisms underlying the approach-avoidance bias is to investigate the role of the involved bodily gesture, i.e., of pushing something away and pulling something towards oneself.^1^

In recent years and decades, an upsurge of embodied theories has been seen in many areas of cognitive science (e.g., Clark, 1998; Gallagher, 2006; Goldman, 2012; Engel et al., 2013). Most of those theories depart from traditional accounts that focus solely on abstract, amodal, symbolic information processing as the basis for cognitive processes (e.g., Fodor, 1975, 1981; Marr, 1982). This development has led to a better understanding of the way in which cognitive processing is shaped by the structure of our body, our bodily actions and/or our interaction with the environment. While it has long been known that appraisals can be modified by performing actions associated with a particular valence (Strack et al., 1988), the exact relationship between an individual’s perceptual and motor representations and the associated approach and avoidance tendencies is still far from clear. One particularly promising embodied account to explain the association between abstract concepts like approach and avoidance and sensorimotor patterns like pushing and pulling, and thereby to an explanation of the automatic approach bias, is the ‘biological meaning model’ presented by Fridland and Wiers (2018).

According to the biological meaning model^2^ (Fridland and Wiers, 2018) the automatic approach bias can be explained by the fact that our body (morphology, physiology, shape and functions) is made up in a certain evolutionarily meaningful way. In particular, the centers of our bodies happen to house the most vulnerable organs. Thus, it seems reasonable for humans to have developed a disposition to only allow trustworthy objects to come close to it. Since those vulnerable regions are crucial to protect, along the same lines it seems reasonable for humans to also have developed a disposition to keep dangerous or harmful objects away from the center. Consequently, according to this model pulling something towards us naturally indicates that it is positive (i.e., trustworthy and nourishing) and pushing something away naturally indicates that it is negative (i.e., dangerous and disgusting).

Unfortunately, while such an embodied account seems *prima facie* plausible, the details are still far from clear. One problem comes from the ambiguity of the connection between movement and meaning. As many authors have pointed out, the interpretation of a movement of the hand away from or towards the body as performed with a joystick is ambiguous (e.g., Chen and Bargh, 1999; Rinck and Becker, 2007; Phaf et al., 2014; Laham et al., 2015). A movement of the hand away from the body can constitute an avoidance-movement in the sense of pushing something away but it can also constitute an approach-movement in the sense of reaching out for something. Along the same lines, a movement of the hand towards the body can constitute an approach-movement in the sense of pulling something towards oneself but also an avoidance-movement in the sense of the withdrawal of one’s hand. And although there is some evidence that there might be a preference of interpretation for the first way (Rinck and Becker, 2007; experiment 1 vs. 2; Neumann et al., 2003), this default interpretation seems to be overridable at least in some cases (e.g., Markman and Miguel Brendl, 2005; Seibt et al., 2008).

Different accounts have tried to resolve the ambiguity. While some have argued that the reference point could be a projection of the self (Markman and Miguel Brendl, 2005), others have criticized this view and shown that an automatic approach bias can even be shown towards an empty box (van Dantzig et al., 2009). Some have characterized the bias in terms of an event-coding account, where fitting features just match together (Lavender and Hommel, 2007; Eder and Rothermund, 2008) and there is no bodily component involved. Others have provided evidence that an interpretation of movement direction cannot be overwritten once the whole body is involved (Eder et al., 2020), and therefore suggested that there is a bodily component involved in the process. Still others have suggested to focus on the change in distance that is achieved by an action (Seibt et al., 2008; Krieglmeyer et al., 2011, 2013). Thus, the type of movement involved, at least as long as it is abstract, has to be interpreted with care.

Regardless of how the controversies regarding all those various accounts and problems turn out, however, there appears to be one fundamental worry that, if justified, would make it difficult to argue—despite the current popularity of embodied approaches to cognition in general and to the automatic approach bias in particular—that the automatic approach bias is in fact embodied at all. The worry is that some studies have reported an automatic approach bias using an approach-avoidance task that just required subjects to press the ↑- and ↓-keys on a keyboard (e.g., Peeters et al., 2012). If an automatic approach bias can occur in the absence of any (significant) bodily approach- or avoidance-movement, no straightforward embodied account can offer an adequate explanation of the underlying mechanisms.

In this paper we try to shed light on this problem and contribute to the current discussion by providing important evidence that the automatic approach bias is indeed embodied or at least carries an embodied component, while at the same time casting doubt on the claim that an automatic approach bias can also be detected by means of button press approach-avoidance tasks. For this we conducted a systematic comparison between two kinds of approach-avoidance tasks. One approach-avoidance task involved a decidedly body-related gesture representing approach and avoidance (pushing vs. pulling using a joystick), the other involved a response movement that is arguably neutral in terms of bodily significance at the very least in the context of approach and avoidance (pressing a button). We used generally positive and negative pictures as stimuli and healthy subjects as trial group. In line with previous research, we expected to find an automatic approach bias for the joystick approach-avoidance task. In light of earlier studies that used button presses (Peeters et al., 2012; Krieglmeyer et al., 2013) we also reckoned with the possibility of finding an automatic approach bias for the button press approach-avoidance task. However, given the recent upsurge of embodied approaches to cognition in general and the promising theories regarding the embodiment of the automatic approach bias in particular (Fridland and Wiers, 2018), we expected that even if an automatic approach bias were to occur in pure button press approach-avoidance tasks at all, actually performing the bodily approach- or avoidance-movement in the joystick approach-avoidance task should potentiate the effect. If we found automatic approach biases in both conditions, and if using a joystick instead of pressing buttons would indeed potentiate the effect, we were ready to argue that the gesture of approach and avoidance that is present in the joystick but not in the button press plays an important role for the explanation of automatic approach biases.

## Materials and methods

### Participants

Fifty-one participants (17 male, all right-handed, mean age of 24 years, standard deviation of ~3.6 years) participated in the experiment. All participants gave written informed consent before the start of the experiment and received either 10€ or course credits in exchange for their participation. All participants had normal or corrected to normal vision and were advanced or native speakers of English. All instructions were shown and explained in English. The Ethics Committee of Osnabrück University approved the study.

### General apparatus

We presented all stimuli on a 24” LCD monitor (BenQ XL2420T; BenQ, Taipeh, Taiwan) with a refresh rate of 114 Hz. The joystick used for the joystick approach-avoidance task (Logitech Gaming Extreme 3D Pro Joystick USB PC, Black, Silver) was directly connected to the computer screen. The response pad used for the button press approach-avoidance task (Black Box Toolkit USB response pad)^3^ was connected to the computer with an extension cable for USB. Matlab’s Psychtoolbox V3 (Kleiner et al., 2007; r2017a; MathWorks Company) enabled us to record reaction times of both the pushing/pulling movements as well as the button presses on the response pad. For the joystick-setup, only the time of initiation of the movement was recorded. The remainder of the movement was not in the focus of our study, since it has been shown that the time of execution does not change over congruent or incongruent approach-avoidance conditions (e.g., Solarz, 1960). Participants positioned themselves in front of the screen such that they could naturally and effortlessly hold and use both devices. This was important to avoid a bias by, e.g., making it hard to push the joystick for participants with short arm length because the device would be too far away. The subjects were instructed to autonomously change between devices when they were requested to do so on the screen. We used MATLAB and R to analyze all data. All data is available online at https://osf.io/k2gc3/.

### Stimuli

**Figure 1:**
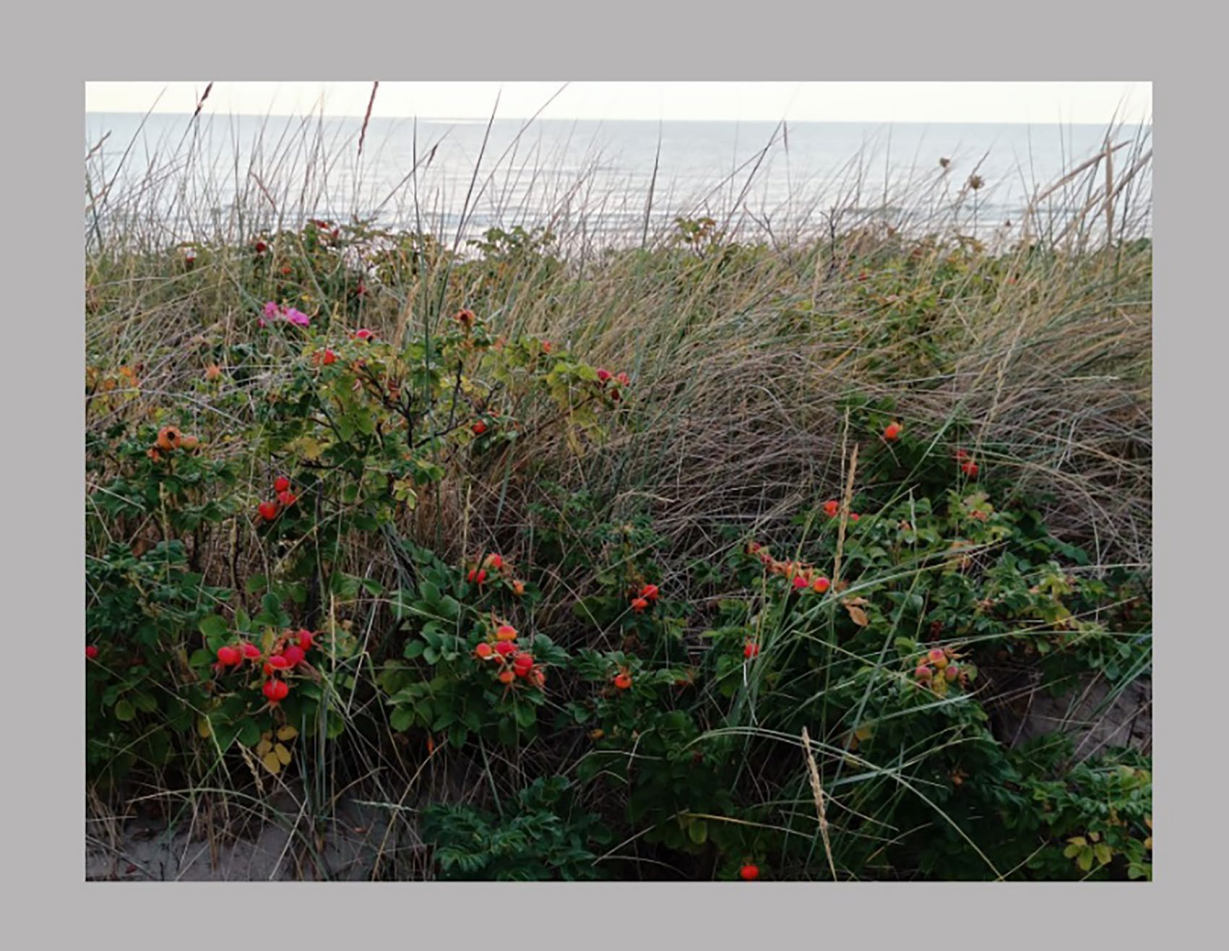
Example-stimulus in its natural resolution in front of a grey background. Note that the picture shown is not actually part of the used stimulus-set to not make IAPS-pictures publicly accessible.

The experiment consisted of four different approach-avoidance tasks for each subject (see below for details). There were two different devices (joystick, response pad) and two different instructions (congruent, incongruent), where participants had to pull positive pictures and push negative pictures for the congruent condition and pull negative pictures and push positive pictures for the incongruent condition. Instruction and device yielded by combination the four different blocks. The first two blocks were always performed with the same device to minimize switches between devices. Both stimuli and block order were randomized over subjects. As stimuli we used 88 full-colored images from the International Affective Picture System (IAPS; Lang et al., 1997). For reasons of comparability we used a stimulus set identical to one being used by other studies and being accessible for other researchers (Kaspar et al., 2015; Czeszumski et al., 2021). Half of the images had a valence rated below 3 (IAPS scale) and served as negative stimuli. The other half had valence ratings above 7.2 and served as positive stimuli. To prevent the images from blurring, we presented all of them in their native resolution of 1,024 × 768 pixels on a grey background (RGB values: 182/182/182), centered in the middle of the screen (resolution of 1,920 × 1,080 pixels; Figure 1).

### Procedure and design

Participants were randomly assigned to one of four groups (A, B, C, D) differing in block order. For convenience we maintained only one switch of devices for each group, while still counterbalancing for both device-order and instruction-order. This yields the following four different sequences (J = Joystick, R = Response pad, I = Incongruent, C = Congruent).

A: JC-JI-RC-RI
B: JI-JC-RI-RC
C: RC-RI-JC-JI
D: RI-RC-JI-JC

In each block, participants faced a sequence of 44 images of different valence (22 positive, 22 negative images). The first four images in each block were test trials and were excluded from analysis. All images were shown twice: Once in the first two blocks and once in the following two blocks. Stimulus order was randomized both between blocks and between subjects. Due to a technical issue the first eight subjects received the same pseudo random stimulus order. Although individuals seem to exhibit an automatic approach bias both for explicit instructions, in which they are instructed to directly react to the valence of the stimuli and for implicit instructions, in which they react to an unrelated feature like stimulus-orientation, explicit instructions seem to be more reliable in producing the automatic approach bias (Phaf et al., 2014). Thus, we instructed participants to react directly to the valence of the pictures, ensuring an affective evaluation of the stimuli. As soon as an image was presented, the participants had to respond to the valence of the image with either the joystick or the response pad, using their dominant hand. In line with previous button press approach-avoidance setups (Peeters et al., 2012) and a recent analysis of the field (Fridland and Wiers, 2018) we assumed that the up button (↑) can naturally be interpreted as pointing away from the participants. Thus, pressing the up button on the response pad corresponded to pushing the joystick (expressing avoidance). Conversely, we assumed that the down button (↓) can naturally be interpreted as pointing towards the subject. Thus, pressing the down button on the response pad corresponded to pulling the joystick (expressing approach). Participants in the congruent condition thus had to pull the joystick towards them or press the down button (approach) whenever a positively valanced image was shown, and push the joystick away or press the up button (avoidance) whenever a negatively valanced image was shown. In the incongruent condition, participants had to act reversely, meaning they had to approach the negatively valanced images and avoid the positively valanced ones. All subjects were instructed to respond as quickly and as accurately as possible. It was not possible to rectify and correct response mistakes. In line with Peeters et al. (2012), we instructed the participants to press the corresponding button on the response pad three consecutive times. We recorded all button presses and evaluated the data using the first button press only. For the sake of completeness and in line with Peeters et al. (2012) we repeated all data analyses also using the third button press without finding significant differences in the results. In line with the inability to correct errors in the joystick task, responses in which different buttons were pressed within one trial were treated as errors.

**Figure 2:**
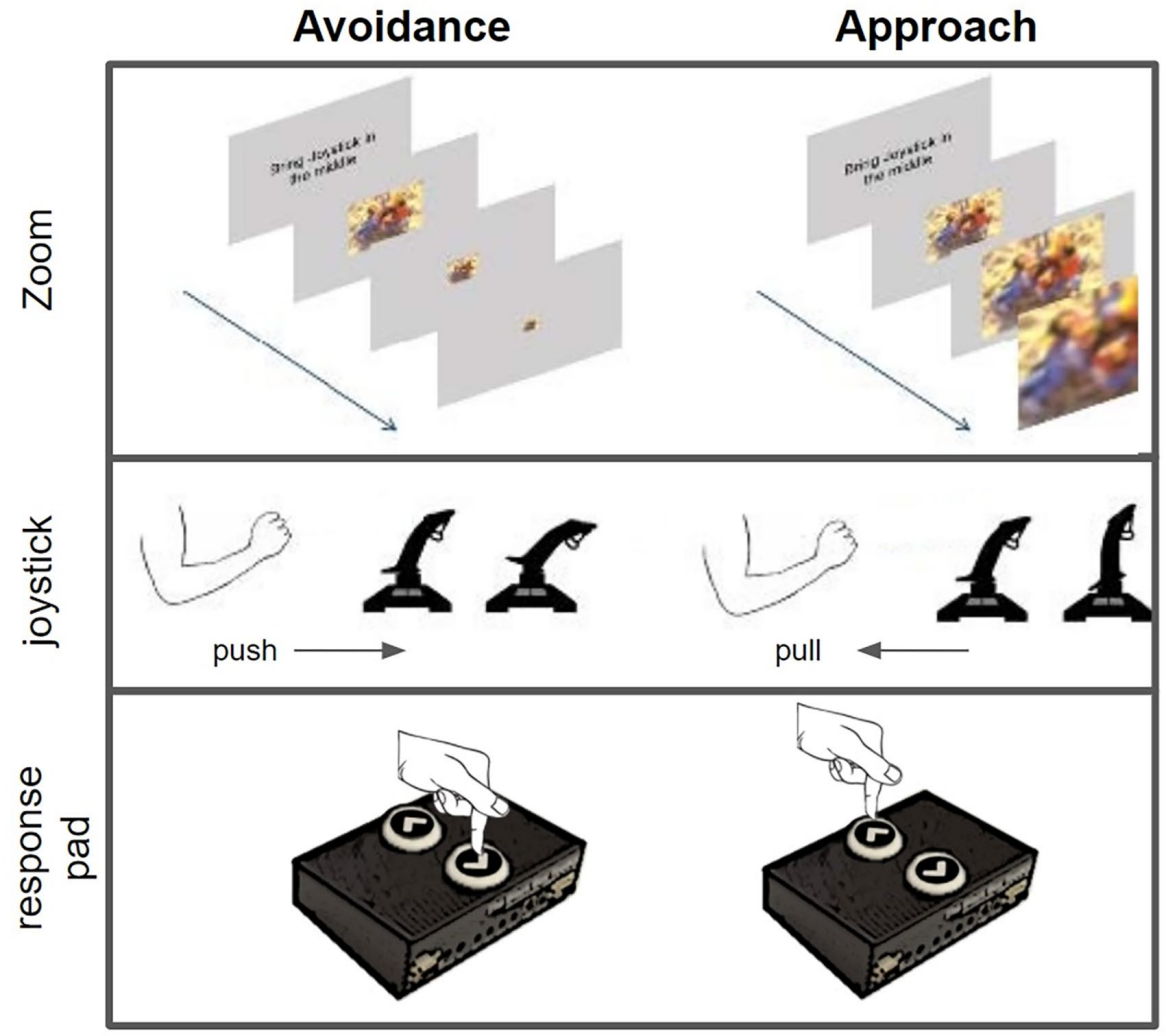
The upper part shows the zoom-effect for both directions. The lower parts show approach and avoidance reaction for both devices (joystick and response pad). This figure is partly reused from Czeszumski et al. (2021).

In line with prior research (e.g., Czeszumski et al., 2021), we used a ‘zoom-effect’ to enhance the impression of a movement of approach or avoidance, respectively. While moving the joystick or when pressing the button on the response pad for the third time, the image changed in size in a way that was supposed to enhance the impression of approach or avoidance. In avoidance-conditions (pushing the joystick, pressing the up button) the image presented smoothly decreased in size. Conversely, in approach conditions (pulling the joystick; pressing the down button) the image presented smoothly increased in size (Figure 2). The zoom effect was initiated with the beginning of the movement and proceeded at a previously fixed speed afterwards. This zoom feature of the approach-avoidance tasks was programmed in MATLAB’s Psychtoolbox V3 (r2017a; MathWorks Company) and taken directly from Czeszumski et al. (2021) with only few adjustments made in the code. Participants were instructed to push or pull the joystick to its limit. Generally, participants had control over when the next stimulus would appear by pressing the index finger button on the joystick or the left button on the response pad. Instructions were repeatedly shown between stimuli to ensure that participants always were aware of the current instruction. Between blocks subjects took a break of at least 15 s.

## Results

### Accuracy and error-trial exclusion

We gathered data from 51 participants * 176 trials, yielding a total of 8,976 data points. Each block started with 4 test trials, which were excluded from analysis (51*4*4 = 816 test trials). Four subjects did not notice a change of instructions between blocks and thus had an accuracy of under 75%. Since those subjects had to be instructed again, they were completely excluded from analysis to avoid instruction biases in the data [4 * (176–16) = 640 additional trials excluded]. For the remaining data (7,520 trials) we calculated an accuracy of 96.3%. Thus, subjects made a low amount of errors (3.7%). This indicates that instructions were clear and all participants remaining in the analysis followed them with high accuracy. Therefore, we excluded all remaining error trials from any further analysis (7,233 trials remaining).

### Pre-processing

To make data accessible and to prepare for statistical analysis in general we decided to perform a pre-analysis observation of the data. First, we divided all data into the four different conditions (joystick-incongruent; joystick-congruent; button press-congruent; button press-incongruent). The raw data showed a right-skewed distribution for all conditions and generally not a lot of variance to the left side of the mean. This is to be expected because the fastest human reaction times have been known to be around 150 ms. Consequently, for values lower than that there is not much space for variance. In contrast, sometimes people needed 3, 4 or even more seconds to react to a picture, allowing for large variance to the right side of the distribution. This distribution of data is characteristic of reaction-time-experiments. However, as Baayen and Milin (2010) have pointed out, the treatment of the ‘tail’ of such a distribution requires a certain amount of caution. Especially the treatment and identification of extreme values is not straightforward. In particular, one important question is whether the effect of interest partly lies in the ‘tail’ of such a distribution or not. We therefore took several measures before we proceeded to the main statistical analysis.

First, all trials with reaction times below 150 ms were identified as not intentional responses but signals unrelated to task and visual stimulation and were discarded (24 trials were discarded this way). Those trials mostly occurred for the joystick condition (all but one trial) and can be explained by subjects putting their hands and therefore weight on the joystick after bringing it back to a neutral position. When subjects then made the next picture appear, the program detected a response immediately because the joystick was in a pulled position already when the picture appeared.

Second, we had to deal with outliers that corresponded to very long reaction times. After the experiment, some subjects reported that they experienced a ‘brain freeze’ for negative pictures they did not want to pull towards them, meaning they came into a state of cognitive dissonance in which they did not want to react at first but then remembered they had to, which led to a delayed reaction. This might be one explanation, among others, for the existence of a few very long reaction times. Statistical analysis of the outliers supported this hypothesis: The further away from the mean, the more extreme values have been found in the incongruent conditions (between 62% for outliers above 2 standard deviations from the mean up to 72% for outliers more than 5 standard-deviations away from the mean). This shows that there might indeed be a connection between the condition (congruent/incongruent) and very long reaction times. This makes it apparent that part of the effect indeed lies in the ‘tail’ of the distribution. However, since reaction times are known to be influenced by a multitude of dimensions like fatigue (Welford, 1968, 1980), age (Welford, 1977) and even breathing cycle (Buchsbaum and Callaway, 1965), their explanation can never be one-dimensional. If part of the effect does lie in the tail of a distribution, it is recommended to not cut of more than 5% of the data (Ratcliff, 1993; Baayen and Milin, 2010). Only around 3% (227 trials) of the data lied outside of 2 standard deviations of the mean. Thus, we decided to use a 2-standard-deviation-winsorizing procedure to adjust the distribution without losing valuable information (reaction times of 227 trials were shifted that way, 46 congruent joystick trials, 91 incongruent joystick trials, 39 congruent button press trials, 51 incongruent button press trials).

Just as for other parametric tests (like ANOVA), linear mixed models also assume the normal distribution of their underlying data. Specifically, for LMMs the residuals of the model have to be normally distributed. Reaction time distributions are generally not normally distributed but belong to the Ex-Gaussian distributions (a convolution of normal and exponential distributions). One way to transform Ex-Gaussian data-sets like reaction time data in such a way that the normality-assumption is met, is to employ a log-transform. For LMMs this means that the model is changed in such a way that it predicts the log of the desired reaction times instead of the reaction time directly. Following this we log-transformed all remaining data before further analysis using natural logarithm (also see Marmolejo-Ramos et al., 2014). Eventually all aspects of the reaction times were treated satisfactorily. We thus continued with the main analysis.

**Figure 3:**
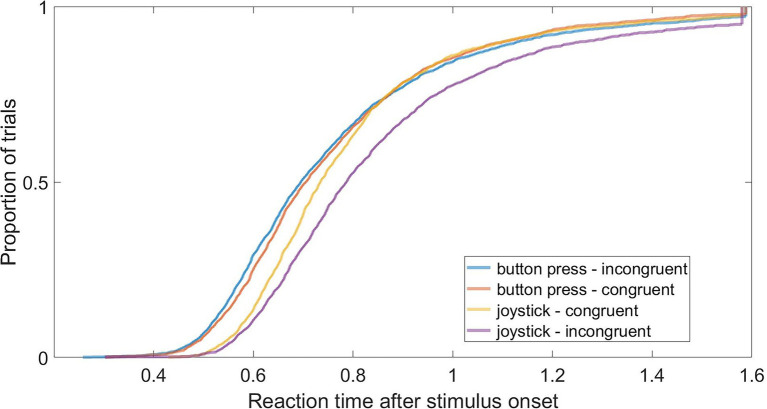
Cumulative Distribution Function of all condition-pairs between joystick/response pad and congruent/incongruent.

All post-preprocessing data is depicted as cumulative distribution functions for all conditions in Figure 3. Note that the data in Figure 3 is not log-transformed for easier readability. The blue and the orange lines (that start on the very left) correspond to the button press conditions, the purple and the yellow lines (that start more to the right) correspond to the joystick conditions. The figure indicates that button press trials are generally faster than joystick trials, as both button press conditions begin to grow significantly earlier and reach their inflection point sooner than the joystick conditions. Additionally, the button press conditions proceed very similarly in their course. However, the joystick conditions do differ in slope. Here, the yellow (the brighter) line that corresponds to the congruent joystick condition shows a steeper slope than the incongruent joystick condition. A steeper slope of a cumulative distribution function indicates that the variance of reaction times is smaller in the respective condition. A smaller variance in the congruent joystick condition in contrast to the incongruent joystick condition indicates that there was higher uncertainty in the incongruent joystick condition than in the congruent joystick condition. Since there is no difference in slope for the button press conditions, there is also no visible variance-difference for those conditions, indicating that there was no difference in uncertainty for button press conditions. Those observations allow for a first pre-analysis interpretation of the data, which suggests that participants were less hesitant in reacting to congruent conditions when using a joystick, but not when using button presses.

### Main effects and interaction

Since our main hypothesis concerned the embodiedness of the automatic approach bias, we focused our analysis on the comparison of the reaction time differences between conditions dependent on the devices used. For all purposes we used linear mixed models (LMM) to analyze reaction times. The LMMs were calculated with the fitlme function of matlab fit by restricted maximum likelihood estimation (REML). Degrees of freedom were assumed to be constant and equal to *n*–*p*, where *n* is the number of observations and *p* is the number of fixed effects (residual method). For the calculation of effect sizes, we used Cohens d. For that we calculated the standard-deviation from the standard-error of the model and we used the specific betas of the model as difference between means. The calculation procedure was the same for all effects. In our LMM we modelled reaction times by condition, device and valence as fixed effects and interactions between them. As random effect we used random intercepts for the grouping variable ‘subject’. The Wilkinson Notation of the model would be:

Reaction Time ~ Device * Condition + (Subject + ε)

For all predictors we used an effect coding scheme with binary factors coded as -0.5 and 0.5. The advantage of this coding scheme is that the fixed effect intercept is estimated as the grand average across all conditions and not as a baseline condition average. Thus, the resulting estimates can be directly interpreted as the main effects. Beside the main effects for ‘device’, ‘condition’ and ‘valence’, for our main hypothesis the interaction between the fixed effects ‘device’ and ‘condition’ were of special interest.

**Figure 4:**
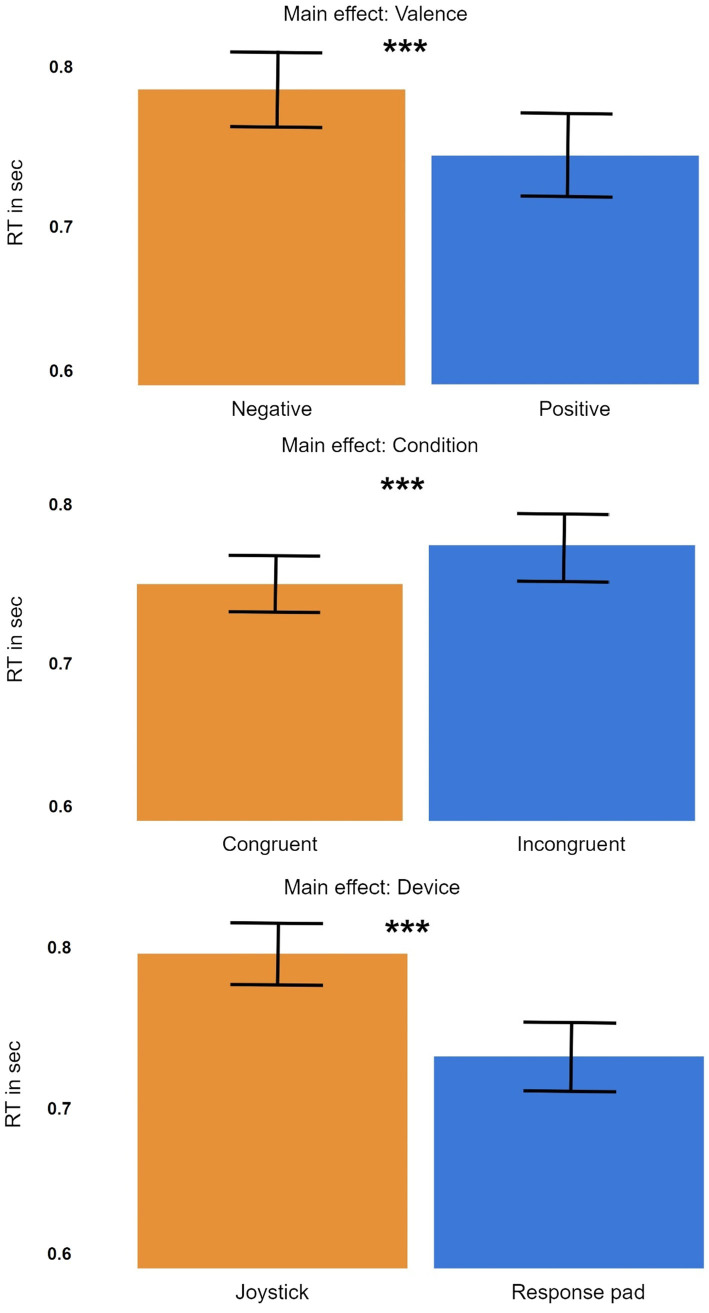
Main effects for device (joystick vs. response pad), condition (congruent vs. incongruent) and valence (positive vs. negative).

First, we found a significant main effect for the fixed effect ‘condition’ (congruent vs. incongruent). The effect was significant with [t(7225) = −6.2569, *p* < 4*10^−10^; Figure 4]. The log-transformed reaction time for the incongruent condition was about 0.038 times higher when compared to the congruent condition (*β* = −0.0334). This corresponds to a percentage-increase in reaction times of 3.3%, meaning participants reacted faster in the congruent condition compared to the incongruent condition by a factor of 3.3%. We calculated an effect size of d = 0.91 for this effect (SE = 0.0053), which is a large effect-size according to Cohen (1988). Averaged over both devices participants were 25.7 ms faster when responding to congruent conditions in comparison to incongruent conditions. Thus, it can be concluded that participants were overall faster in responding in congruent than in incongruent trials.

Furthermore, we also found a significant main effect for the fixed effect ‘device’ (joystick vs. button press). Here, the effect size was significant with [t(7225) = 15.584, *p* < 7*10^−54^; Figure 4]. The log-transformed reaction time for button presses on the response pad was about 0.083 times smaller than for the joystick condition (*β* = 0.0833). This corresponds to an increase of 8.7% between devices, meaning participants were 8.7% faster for button press trials compared to joystick trials. We calculated Cohens d for the main effect ‘device’ with d = 2.27 (SE = 0.0053), which corresponds to a large effect size (Cohen, 1988). Here, averaged over both conditions participants were 64.4 ms slower when responding with a joystick in comparison to responding with the response pad. It can be concluded that averaged over both conditions participants had slower reaction times for the device ‘joystick’ in comparison to the device ‘response pad’.

Lastly, we found a significant main effect for the fixed effect ‘valence’ (positive vs. negative). The effect was significant with [t(7225) = −10.082, *p* < 10^−24^; Figure 4]. The log-transformed response time for negative pictures was about 0.054 times smaller than for positive pictures (*β* = −0.0539). This corresponds to a reaction time increase of 5.25% between positive and negative pictures. Thus, participants were 5.25% faster in responding to negative pictures compared to responding to positive pictures. Cohens d for the main effect ‘valence’ was calculated with d = 1.47 (SE = 0.0053), which is a large effect size (Cohen, 1988). It can be concluded that participants were significantly faster in reacting to negative stimuli than in reacting to positive stimuli.

**Figure 5:**
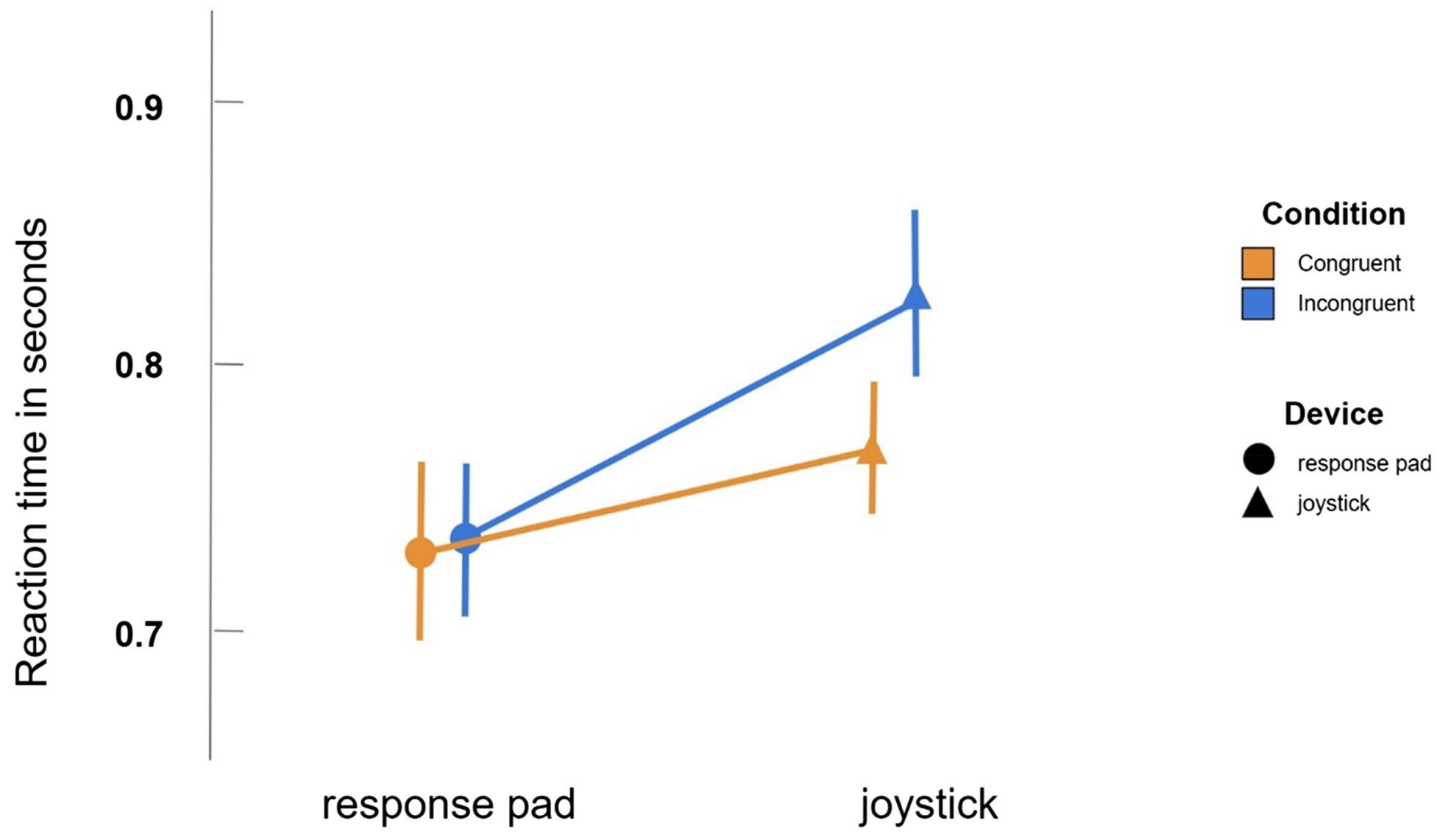
Interaction between condition and device. Participants were significantly slower for the incongruent condition when using the joystick but not when using the response pad. Note that the standard error in the figure suggests a bigger divergence than the actual LMM. This is because the model uses an inter-subject comparison (random variable ‘subject’), but the standard error does not.

We found a significant interaction between the fixed effects ‘device’ and ‘condition’ (Figure 5). This interaction was significant with [t(7225) = −7.3452, *p* < 2*10^−13^]. All other interactions were tested, but no other two or three-way interaction was significant (all *p* > 0.20). Means and standard error of the mean for the four conditions were: JC: 0.7695 s (SEM: 0.0256), JI: 0.8274 s (SEM: 0.0297), BC: 7316 (SEM: 0.0283), BI: 0.0.7355 s (SEM: 0.0328). The difference in reaction time between congruent and incongruent condition was larger for the joystick than for the button press by a factor of 14.8. The difference in reaction time between joystick and button press was larger for incongruent conditions than for congruent conditions by a factor of 2.8. Consequently, the longest average reaction time was observed for the incongruent joystick condition. Cohens d for the interaction ‘device: condition’ was calculated with d = 1.07 (SE = 0.011), which still corresponds to a large effect size (Cohen, 1988). As Figure 5 displays, the interaction between the parameters device and condition explains a lot of the main effects. While button presses are generally faster than joystick trials and congruent trials are generally faster than incongruent trials, it really is the incongruent joystick condition that makes the difference. The interaction shows that incongruent trials are significantly slower than congruent trials when using a joystick, but there is merely a difference between them when using a response pad.

## Discussion

In the present study we tried to resolve the problem that despite its theoretical (and potentially also clinical; see footnote 1) potential, an embodied take on the automatic approach bias seems hard to sustain if such a bias is present in a setup that requires no significant bodily behaviour connected to approach or avoidance to begin with Fridland and Wiers (2018). Therefore, we conducted a systematic comparison between two different approach-avoidance tasks, one using button presses and one using a joystick. By this we aimed at investigating whether the gesture in the sense of an actual bodily movement influences—or might even be necessary for — the effect. In line with previous research we expected to find a main effect for the joystick (Phaf et al., 2014) and for the button press condition (Peeters et al., 2012). Due to recent considerations of the embodied paradigm regarding approach and avoidance gestures (Fridland and Wiers, 2018) we also hypothesized that the effect would be more pronounced for the joystick than for the button press, and that this difference could be explained by the difference in device.

The following discussion concerns the interpretation of the different main effects we found (valence, device, condition) and of the significant interaction between device and condition. We found strong main effects for all fixed variables (valence, condition, device). With those findings we reproduced known effects of the field.

*First*, we reproduced the finding that negative stimuli are generally faster responded to than positive stimuli. This can be explained by the evolutionary necessity to act faster when confronted with something negative (i.e., dangerous) and confirms theories about different pathways of processing (e.g., Lang et al., 1990).

*Second*, we reproduced the general effect of an approach avoidance bias, meaning subjects being faster in approaching positive stimuli and avoiding negative stimuli rather than vice versa (e.g., Czeszumski et al., 2021).

*Third*, we found a main effect for device that suggests that button presses are generally faster than joystick trials.

As Figure 5 shows, both main effects (device and condition) are mostly explained by the strong divergence between congruent and incongruent trials for the joystick task. This shows that participants were slower for incongruent trials only if they used a joystick, but not when they used the response pad. Since the major difference between those devices is supposed to be the gesture involved, it can be concluded that the gesture does indeed play a crucial role in the origination of the approach-avoidance bias. That the automatic approach bias is more complex than originally thought, was already pointed out by Phaf et al. (2014), who emphasized the role of different types of instruction and context. With the described findings and the resulting importance of the involved gesture, this study adds another layer to the complexity of automatic tendencies of approach and avoidance.

Although, e.g., Peeters et al. (2012) did report an automatic approach bias only using button presses, we could not reproduce those findings. This could have several reasons. First, it could be that an automatic approach bias for button presses is just weaker than for joystick tasks. In that case, a too small sample size could have prevented us from finding the effect. If this was the case, our results still show that a supportive gesture potentiates the effect. Second, it is conceivable that there simply is no effect for button press approach-avoidance tasks and that the former findings could not be reproduced due to the nonexistence of the effect. In this case, the gesture would not only potentiate an existent effect, but would be necessary for its existence. Third, we might have been unable to reproduce the effect due to other differences in the setup. For example, as Phaf et al. (2014) already suggested, the role of affect in this context is yet unclear. Peeters et al. (2012) were working with at risk subjects concerning alcohol abuse and it might be that the general effect is stronger in psychopathological contexts in contrast to working with healthy subjects. If and how exactly such an effect modifies the automatic approach bias has yet to be shown in further research. Fourth, we might have been unable to show an effect for button presses due to a floor-effect, meaning that participants have been so fast in pressing the button on the response pad that differences between valences were not or only barely observable. Our data, however, shows that in the response-pad-button condition not only the mean of the reaction time is unchanged but the cumulative distribution functions aggregated over all trials are virtually identical (Figure 3). That is, even the fraction of trials with rather long reaction times is not reduced. This makes a general floor-effect an unlikely interpretation. Fifth, it could be conceivable that approach-avoidance tendencies emerge over time and as a reaction to the zoom effect. Since the button press zoom only appeared after the third button press, while the zoom of the joystick appeared immediately after initiation of the movement the reinforcement might work for the joystick but not for the button press. This seems unlikely, however, since the time interval between the three button presses is rather short. Furthermore, other studies have shown approach-avoidance effects completely without zooming-effects before (Solarz, 1960; Chen and Bargh, 1999; Rotteveel and Hans Phaf, 2004). Sixth, just as there is an ambiguity for joystick-movements, there might also be an ambiguity regarding the interpretation of up and down button presses. While the up button points away from the subject, indicating avoidance, it could also be interpreted as showing towards the stimulus, indicating an approach behaviour of the subject. Likewise, the down button points towards the subject, indicating approach behaviour, but could also be interpreted as pointing away from the stimulus, and thus indicating an avoidance movement. It might be that this double-edged interpretation negates an effect that could otherwise be measured. However, this ambiguity in interpretation seems to be grounded in the question of the reference frame, meaning the question what is the thing that gets moved. If it is the subjects that moves, then an up button might suggest approach, moving the subject towards the stimulus. If it is the stimulus that is moved, an up button should suggest avoidance, moving the stimulus away from the subject and vice versa for the down button. Fortunately, the zoom effect should make the reference frame clear by zooming in and out the *object*. Thus, it seems unlikely that this ambiguity led to the absence of an effect for the button presses.

It can be concluded that while our results suggest that the gesture involved in approach avoidance tasks plays a role for an automatic approach bias to emerge, it is not clear yet how exactly it contributes to the bias, i.e., whether it potentiates an existent effect or is even necessary for the effect’s existence or plays yet another role.

We demonstrated that the gesture—the movement of the arm away or towards our body—in a situation of approach and avoidance matters. The following will focus on what this *means* for theories of embodiment. If we push something away, we do not want it to be close to us, we avoid it; if we move something towards our body, be it something to eat or a person that we want to hug, this is a movement of approach. The movement of our arm—the gesture that we use to approach or to avoid certain things in our environment—supports our action. The observation that certain compatibility effects—like the automatic approach-avoidance bias—are only present or at least stronger if combined with a corresponding gesture suggests that there is more to the gesture than it just being a part of the process of approach and avoidance. It suggests that the gesture is part of what it means to approach or avoid something as Gallagher (2006) and many other authors have claimed. In this sense the automatic approach bias is embodied, possibly in one of the ways that Fridland and Wiers (2018) suggested.

Yet, there is more to gestures. While the idea that pushing something away from us means avoidance and pulling something towards us means approach, this cannot be the full story. If one gets scarred and withdraws his/her hand from a spider this is a movement towards the body while at the same time being an avoidance-movement. As seen above, another factor to consider is the reference frame: A joystick movement does not necessarily correspond to a natural movement in a neat one-to-one fashion. If one pushes the joystick away, that might as well be interpreted as a movement of one’s body into the same direction (as, for example, in videogames, when controlling an avatar). For example, in manikin-tasks or modified approach avoidance tasks, corresponding biases have been found, if the context was different for the participants. Thus, it seems that tendencies of approach and avoidance and their corresponding gestures are context-sensitive (e.g., Markman and Miguel Brendl, 2005; Zhang et al., 2012). Further research might show how context sensitivity influences the embodiment of the automatic approach bias.

Lastly, if, as our data suggests, the automatic approach bias is indeed embodied, this might have clinical implications. For there is at least some evidence that implicit approach-avoidance tasks performed over a longer period of time in which participants have to push adverse cues away while pulling neutral cues towards them can erase or reverse an automatic approach bias, for instance in individuals struggling with a substance use disorder, other addictions, and psychological disorders. Quite generally, by means of such a process of ‘cognitive bias modification’ individuals can be brought to lose their initial tendency to approach, say, drug-related stimuli faster than they avoid them, or to avoid feared objects faster than they approach them, with a positive effect on their success of treatment, craving rates, subconscious action tendencies etc. (e.g., MacLeod and Mathews, 2012; Rabinovitz and Nagar, 2015; Wiers et al., 2015; Mühlig et al., 2016; Lindenmeyer, 2019; Mehl et al., 2019). To the extent that such cognitive bias modifications can be achieved by means of joystick approach avoidance tasks, our results that indicate that the gesture itself has an influence on the strength of the effect suggest further speculations.

If the modification of the automatic approach bias indeed ‘scales up’ with stronger gestures, then joystick-based cognitive bias modifications should be more efficient in modulating the automatic approach bias than cognitive bias modifications using less pronounced bodily movements such as button presses. Furthermore, this modulation should scale up with even more intense and ecologically valid gestures. If even a relatively ‘weak’ gesture like a joystick movement—that indeed is associated with approach and avoidance but far from really *incarnating* the concepts—can have a strong effect on changing the reaction-time-bias of a person, it might be possible to get an even stronger difference by enlarging the immersion or choosing even stronger gestures, that really are *incarnating* the concepts of approach and avoidance: Imagine, say, a subject pushing away an object of avoidance, like alcohol with a full-body movement and both hands. Similar ideas could work for approach when subjects suffering from phobia make full body-movements towards the object of fear. The corresponding cognitive bias modifications could be implemented into VR to additionally increase the immersion. This might ultimately yield even stronger effects on reaction-time changes. Obviously, these exploratory ideas must be treated with caution and should be tested and arranged with the help of psychotherapists to avoid any danger for the subjects. One worry could be that full body movements of avoidance also might elicit anger, which should not be involved in the therapy of patients suffering from alcohol abuse disorder what so ever.

Furthermore, even the question whether effects found in mere reaction-time setups can translate into long-term behavioural changes for subjects suffering from psychiatric disorders is not yet resolved. Cristea et al. (2015) argued that there might be only small behavioural effects of such modifications, but that it might also be that there are no effects at all: A lot of the studies that proclaim to show such success, they point out, suffer from small or low-quality-trials and publication bias might play a major role in this field. That being said, Cristea et al. (2015) only consider studies on anxiety and depression and the most prominent studies on cognitive bias modifications have been proclaimed in other areas such as alcohol use disorders (e.g., Wiers et al., 2011) and more recent meta-analyses (e.g., Kakoschke et al., 2017; Batschelet et al., 2020) and randomized clinical studies (e.g., Manning et al., 2021) in these areas are more optimistic than Cristea et al. (2015). Moreover, even if the effects on behavioural parameters might be small, this might partly be due to the under-development of the treatment, e.g., as a mere add-on-therapy or ‘homework’ for patients between their sessions. If it should, however, turn out that reaction time-changes in cognitive bias modifications do actually translate into behavioural changes, or even just into a disposition of the patients to change their behavior more easily, then increasing the immersion and choosing stronger gestures might be a way to improve the concept.

In the end, a lot of questions regarding an embodied approach to cognitive bias modifications are still unanswered. The mental and physical health of patients suffering from serious psychiatric disorders must always have highest priority. Because of that, every possibility to help those should be explored, including cognitive bias modification and its improvement. We show one dimension that might be a way to alter those concepts. Promising trials like Eiler et al. (2019) demonstrate a first implementation of the concept for smokers and ensure the potential and the worth of investigating into this direction.

## Author’s note

A stable link to a repository with the data can be found in the Data availability statement. This study was not preregistered.

## Data availability statement

All raw data supporting the conclusions of this article are available at: https://osf.io/k2gc3/.

## Ethics statement

The studies involving human participants were reviewed and approved by the Ethics Committee of Osnabrück University. The patients/participants provided their written informed consent to participate in this study.

## Author contributions

SW and JS contributed to the idea of the study. JS and PK contributed to conception and design. JS contributed to the data collection of the study and wrote the first draft of the manuscript. AC and JS performed the statistical analysis. All authors contributed to the article and approved the submitted version.

## Funding

This project was funded by the Deutsche Forschungsgemeinschaft (DFG, German Research Foundation)—project number GRK-2185/1 (DFG Research Training Group Situated Cognition) and the Deutsche Forschungsgemeinschaft (DFG) Open Access Publishing Fund of Osnabrück University.

## Conflict of interest

The authors declare that the research was conducted in the absence of any commercial or financial relationships that could be construed as a potential conflict of interest.

## Publisher’s note

All claims expressed in this article are solely those of the authors and do not necessarily represent those of their affiliated organizations, or those of the publisher, the editors and the reviewers. Any product that may be evaluated in this article, or claim that may be made by its manufacturer, is not guaranteed or endorsed by the publisher.

## Footnotes

^1^Identifying the mechanisms underlying the approach-avoidance bias might be particularly important given that some have recently considered the possibility of using approach-avoidance tasks for helping individuals struggling with substance use or other forms of psychiatric disorders (see ‘Discussion’).

^2^Besides the biological meaning model Fridland and Wiers also offer a second embodied account. It is called ‘sensory motor hypothesis’ and reflects the idea of approach and avoidance as learned sensory motor loops.

^3^ https://www.blackboxtoolkit.com
